# Prediction of Temporal Liking from Temporal Dominance of Sensations by Using Reservoir Computing and Its Sensitivity Analysis

**DOI:** 10.3390/foods13233755

**Published:** 2024-11-23

**Authors:** Hiroharu Natsume, Shogo Okamoto

**Affiliations:** Department of Computer Science, Tokyo Metropolitan University, Hino 191-0065, Japan; natsume-hiroharu@ed.tmu.ac.jp

**Keywords:** sensory evaluation, temporal dominance of sensations, temporal liking, machine learning, reservoir computing, strawberry

## Abstract

The temporal dominance of sensations (TDS) method has received particular attention in the food science industry due to its ability to capture the time–series evolution of multiple sensations during food tasting. Similarly, the temporal liking method is used to record changes in consumer preferences over time. The conjunctive use of these methods provides an effective framework for analyzing food taste and preference, making them valuable tools for product development, quality control, and consumer research. We employed the TDS and temporal liking data of strawberries that were recorded in our earlier study to estimate the temporal liking values from sensory changes. For this purpose, we used a reservoir network, a type of recurrent neural network suitable for time–series data. The trained models exhibited prediction accuracy of the determination coefficient as high as 0.676–0.993, with the median being 0.951. Further, we proposed two types of sensitivities of each sensory attribute toward the change in the temporal liking value. Elemental sensitivity indicates the degree that each sensory attribute influences the temporal liking. In the case of strawberries, the sweet attribute was the greatest contributor, followed by the attribute of fruity. The two least-contributing attributes were light and green. Interactive sensitivity indicates how each attribute affects the temporal liking in conjunction with other attributes. This sensitivity analysis revealed that the sweet attribute positively influenced the liking, whereas the green and light attributes impacted it negatively. The proposed methods offer a new approach to comprehensively analyze how the results of TDS are linked to those of the temporal liking method, serving as a step toward developing an alternative system to human panels.

## 1. Introduction

The temporal dominance of sensations (TDS) method is widely used to track how multiple sensations evolve over time during the tasting of foods or beverages [1,2,3]. Before the emergence of this method, simultaneously tracking multiple types of sensations was limited [4,5]. By recording the timing and duration of dominant sensations, the TDS method provides a detailed understanding of the dynamic nature of sensory experiences (e.g., [6,7,8]) and is effective in distinguishing between different brands (e.g., [9]).

Since capturing the temporal transitions of sensations is a key aspect of the TDS method, the results of TDS experiments are typically visualized through TDS curves [2,10] and trajectory plots [11,12,13]. Additionally, various graphical and statistical approaches are available for deeper analysis [14,15,16,17]. For example, the panel behavior in TDS tasks has been modeled as a probabilistic state transition using semi-Markov chains [14,16].

More recently, the application of TDS has expanded beyond food stimuli, finding use in fields such as cosmetics evaluation and food tasting tasks paired with music [18,19,20,21]. These advancements suggest that TDS can serve as a general method of sensory evaluation in domains beyond food science.

The temporal liking method, on the other hand, records time–series data on food preferences or perceived deliciousness [22,23]. This method originated from early studies conducted in the 1990s [5]. Unlike traditional evaluations, which typically assess overall liking after consumption, the temporal liking method captures real-time changes in preference during the tasting process. The results are visualized through a time–series curve, known as the temporal liking curve.

Integrating the TDS and temporal liking methods has the potential to significantly advance sensory evaluation techniques. Several studies have attempted to connect these two methods. For instance, Thomas et al. [22] proposed analyzing the average temporal liking score when specific sensations were dominant, which is known as LWD, allowing for a joint analysis of both methods. Thomas Carr et al. [24] explored how well the modified LWD values distinguish six different types of cheeses. Meyners [23] superimposed differential temporal liking curves onto differential TDS curves to visually identify key drivers of food preference. He also divided the tasting period into smaller intervals, analyzing TDS data and temporal liking within each segment as static, non-time–series data to compute the influences of TDS attributes on the liking score. Castura et al. [12] examined how transitions between dominant attributes were linked to the average temporal liking score at the moment those transitions occurred. Ares et al. [25] compared the TDS method with the temporal check-all-that-apply method [26] to determine which better explains impact on consumer preferences, as reflected in temporal liking. Silva et al. [27] investigated the ability to differentiate between wines with similar flavor profiles using multiple methods, including TDS and temporal liking. Nguyen and Varela [28] identified sensory attributes that influence temporal liking scores at various time points during yogurt tasting. While these studies primarily contrasted TDS and temporal liking data, research connecting the TDS method to overall liking scores also presents a promising approach for understanding the drivers of food preference [29,30,31].

Despite these advances, no studies have directly estimated temporal liking curves from TDS curves. Statistical or machine learning techniques are effective for this purpose; however, they require substantial datasets to function effectively. Conducting sensory evaluation tasks is time-consuming and costly, and typical TDS and temporal liking tasks do not generate sufficient data for data-driven prediction techniques. Specifically, TDS and temporal liking tasks generally produce a single set of averaged tasting experiences for each consumer group, which is not ideal for machine learning approaches.

This limitation can be addressed using data augmentation or resampling techniques, such as bootstrap resampling [9,32]. This method expands the dataset while preserving its statistical variability. For instance, bootstrap resampling can estimate the distribution of TDS curves, enhancing the dataset’s usability for predictive modeling [9,13,32].

Developing a method to predict temporal liking curves offers two key advantages. First, it provides a cost-effective alternative for sensory evaluation. If temporal liking can be reliably predicted from TDS curves, the separate temporal liking task could be eliminated, significantly reducing costs. However, as this study is in the feasibility proof stage, the exact percentage of temporal liking tasks that could be saved remains unclear. Second, this approach enables a detailed analysis of how specific sensory attributes influence temporal liking, offering deeper insights into the relationship between the two curves. By modeling these relationships, we can gain a clearer understanding of how individual sensations shape overall preferences.

In this study, we use reservoir computing, a machine learning framework designed for processing time–series data, which is particularly well-suited for capturing temporal dependencies and patterns [33]. Reservoir computing utilizes a recurrent neural network with fixed internal weights, requiring only the output layer to be trained, making it computationally efficient. While machine learning has rarely been applied to model the relationship between TDS and temporal liking, our previous work [34] demonstrated the potential of reservoir computing for this task, showing that it can effectively predict temporal liking based on TDS curves. In the current study, we employ a cross-validation approach to rigorously evaluate the predictive capabilities of reservoir computing.

Furthermore, we introduce two types of sensitivity analyses to quantify the influence of sensory attributes recorded through TDS on temporal liking. These analyses include elemental sensitivity, which measures the independent contribution of each attribute, and interactive sensitivity, which examines how attributes interact to influence preferences. By combining predictive modeling with sensitivity analyses, we aim to provide a comprehensive method for understanding how sensory attributes drive consumer preferences.

## 2. Temporal Dominance of Sensations Method and Temporal Liking Method

In this study, we used the data of strawberries collected in our previous study [34]. Here, we reviewed the TDS and temporal liking tasks and obtained data.

### 2.1. Food Samples: Strawberry

In each task, participants consumed a whole strawberry in a single bite. The strawberries used for the tasks were of the Tochi-aika variety. Fully ripe strawberries were purchased from a supermarket on the day of the experiment, with each piece costing an average of 120 JPY. The strawberries were served at an air-conditioned room temperature of approximately 23 °C.

### 2.2. Participants

Thirty-one university students participated in the experiments, each completing the TDS and temporal liking tasks three times. The sample size was determined based on the recommended number of TDS samples suggested in [32]. None of the participants were majoring in food sciences or employed in food-related industries. Prior to the main tasks, participants practiced performing the two types of tasks using the computer application, without tasting actual food samples. This practice session was designed to familiarize them with the task procedures and the arrangement of buttons on the interface. The order of the two tasks was counterbalanced across participants. The experiments were conducted at least three hours after participants had their last meals. All participants provided written informed consent before participating in the experiments.

### 2.3. Ethical Statement

The experimental protcol was approved by Institutional Review Board, Hino Campus, Tokyo Metropolitan Uniersity (#R6-008).

### 2.4. TDS Tasks

The experiment using the TDS method was conducted with a user interface displayed on a computer screen. The interface featured a start/stop button and buttons labeled with attribute words. Following the study by Shimaoka et al. [35], where strawberries were evaluated using the TDS method, eight attributes were included: aromatic, juicy, sweet, fruity, light, watery, green, and sour. The descriptions of the attributes [35] (Table 1) were provided to the participants (panels) in advance.

The participant began the task by pressing the start button as soon as the food sample was placed in their mouth. They then tasted the sample to evaluate its sensory qualities. During the task, the participant sequentially pressed the buttons corresponding to the attribute that best described the sensation they perceived as dominant at that moment. The selections reflected the attributes that came to mind rather than their intensities. The participant switched buttons whenever the dominant sensation changed, and multiple attributes could not be selected simultaneously. Some attributes may not have been selected at all during the task. Once the food sample was swallowed, the participant ended the task by pressing the stop button. Each task lasted approximately 20–30 s. Participants rinsed their mouths with water before proceeding to the next trial.

### 2.5. Resutls: TDS Curves

TDS experimental results are typically visualized as TDS curves [1,2], with each curve representing the time–series evolution of the dominance proportion of an attribute. The dominance proportion is defined as the percentage of trials in which a given attribute was selected at a specific time point. This proportion is calculated across all participants and trials. As a result, at each time point, the sum of proportions for all attributes equals 1.0, except during the very early phase of the task, when some participants may not select any attributes immediately after pressing the start button.

To calculate the dominance proportions, the time axis of each trial was normalized to a scale from 0 (when the start button was pressed) to 1 (when the stop button was pressed). This dimensionless time normalization enabled the analysis of average sensory experiences, even when tasting duration varied among individuals. We did not exclude trivial curves that did not reach the chance level or significant proportion, as outlined in the guidelines of [1].

**Figure 1:**
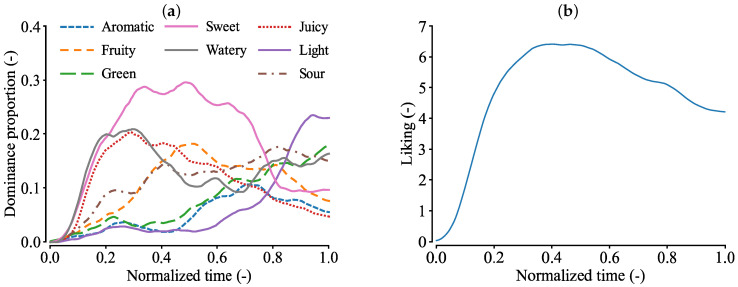
TDS and temporal liking curves of strawberries. Adapted from [34]. (**a**) TDS curves of strawberries: in the early phase, sweet, juicy, and watery sensations were the most prominent, while light and sour sensations became dominant in the final phase. (**b**) Temporal liking curve of strawberries: participants rated the strawberries highest at the midpoint of the task.

Figure 1a shows the TDS curves for strawberries. In the early phase, sweet, juicy, and watery attributes were perceived as dominant, while sour became dominant in the latter phase. These are typical TDS curves for strawberries [7,35], although they differ from those for strawberry pulps [36].

### 2.6. Temporal Liking Tasks

Similar to the TDS method, participants used a graphical interface to evaluate their temporal preference for strawberries. The computer screen displayed nine buttons, numbered from 1 (least) to 9 (most). The procedure for starting and ending the task was identical to that used in the TDS method. During the task, participants rated their current preference for the strawberry in their mouth by pressing the appropriate button. They re-selected buttons whenever their preference changed. As with the TDS task, each session lasted approximately 20–30 s.

### 2.7. Results: Temporal Liking Curves

Figure 1b shows the temporal liking curve for strawberries, which represents the average liking scores across all participants. The score peaked between the normalized times of 0.3 and 0.5. Note that the score is below 1.0 immediately after the task began, as some participants did not rate their preference at the start, and their scores were recorded as 0.

## 3. Reservoir Model for TDS and Temporal Liking Curves

### 3.1. Echo State Network

**Figure 2:**
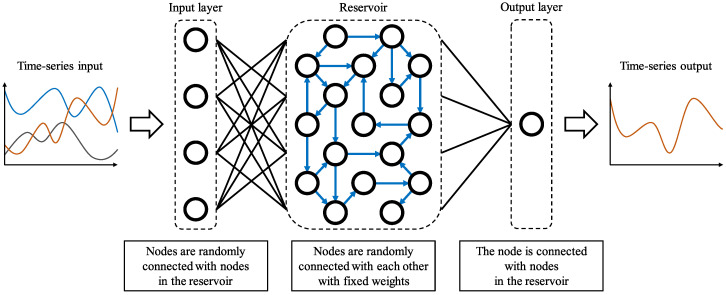
ESN Model. the input layer is connected to the reservoir, where the weights between nodes are fixed and randomly distributed. In the reservoir, nodes are randomly connected to each other with fixed, randomly distributed weights. The output layer is connected to the reservoir, and its weights are adjusted during training.

We utilized an echo state network (ESN) [33], a widely used model in reservoir computing. The model was implemented using ReservoirPy (version 0.3.11) [37], a Python library designed for reservoir computing. As illustrated in Figure 2, the ESN consisted of three layers: an input layer, a reservoir, and an output layer. The time–series data of predictors were fed into the input layer. In this study, the ESN received the dominance proportions of eight sensory attributes as input. The reservoir layer, a recurrent neural network, retained information about past inputs, functioning as a form of memory. The output layer generated the time-series of temporal liking.

At each time step, the ESN processed the eight TDS values and output a corresponding temporal liking value. Since the output at any given moment was influenced by the input history, the ESN mimicked the process by which humans evaluate food preferences over time.

The reservoir was a randomly connected recurrent neural network with fixed connections and weights. During training, only the weights between the reservoir and the output layer were adjusted, allowing for faster learning.

The input layer was configured with eight nodes (or dimensions) to accommodate the TDS curves of the eight sensory attributes. The output layer had a single dimension, as the liking score was represented by a scalar value. The number of neurons in the reservoir was set to 128. We evaluated the model’s prediction accuracy by varying the number of neurons from 60 to 200, ultimately selecting 128 as the optimal number based on the root mean squared error (RMSE), as described in Section 3.4.

A key advantage of the ESN is its nonlinear nature, which enables high-performance prediction. However, this advantage comes with a challenge: difficulty in interpreting why the ESN produces certain outputs, a limitation commonly referred to as a lack of explainability. The sensitivity analysis presented in Section 4 is an attempt to partially address this issue by quantifying the impact of specific input attributes on the model’s predictions.

The ESN is not the only method available for predicting temporal liking curves from TDS curves. For instance, the state-space model also possesses memory functions that make it well-suited for handling time–series data. Although this linear method has been applied to TDS curves for different purposes [38,39], its linear nature allows for easier interpretation of the roles of memory states, facilitating an understanding of the relationship between inputs and outputs.

Despite its interpretability, the state-space model may struggle to capture nonlinear relationships between TDS data and temporal liking, which are critical for accurate predictions. The ESN, by contrast, is specifically designed to handle such nonlinearities, making it a better fit for this task. While we did not directly compare the predictive performances of these methods, we believe the ESN’s ability to model complex dynamics justifies its use in this study. However, the state-space model remains a viable alternative and may be preferred in scenarios where interpretability is prioritized over predictive power.

### 3.2. Bootstrap Resampling

To generate the datasets for training and validating the ESN, we applied bootstrap resampling [9,32] to both the TDS and liking curves. Bootstrap resampling is a statistical technique that estimates the population parameters by randomly drawing observations, with replacement, from the original data.

First, we calculated a single TDS curve set per participant, each of whom repeated three TDS trials. Similarly, the liking curves were averaged for each participant. This process yielded 31 pairs of TDS and liking curves. From this set of 31 samples, we extracted a new sample set of 31, allowing for the overlap of samples, and averaged the pairs of TDS and liking curves to form a single dataset. Thus, a single dataset includes the TDS curves of eight attributes and one temporal liking curve. By repeating this resampling process, we generated 1600 training datasets and 160 validation datasets for each model.

### 3.3. Established ESN

We trained 16 different models using different datasets. Each model was trained with 100 curve sets and validated with 10 curve sets, both generated through bootstrap resampling. Each model was trained on a unique set of curve sets. The hyperparameters for each model were adjusted to minimize the loss, measured by RMSE. The ranges of the model parameters are provided in Table 2. For detailed explanations of these parameters, refer to the literature [33,40].

### 3.4. Prediction Performance

**Figure 3:**
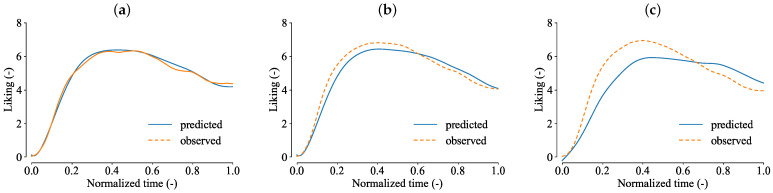
Predictions of temporal liking in strawberry eating experience. (**a**) Prediction with the best accuracy (RMSE=0.137 and $R^{2}=0.993$). (**b**) Prediction with the median accuracy (RMSE=0.386 and $R^{2}=0.951$). (**c**) Prediction with the worst accuracy (RMSE=0.941 and $R^{2}=0.724$).

The 16 models estimated the temporal liking curves using 10 different sets of TDS curves for validation. The range of coefficients of determination was $0.676\leq R^{2}\leq 0.993$ (median: 0.948), and the range of loss was 0.137≤RMSE≤0.941 (median: 0.386). Figure 3 shows the observed and predicted liking curves for the cases with the best, median, and worst prediction accuracy.

**Figure 4:**
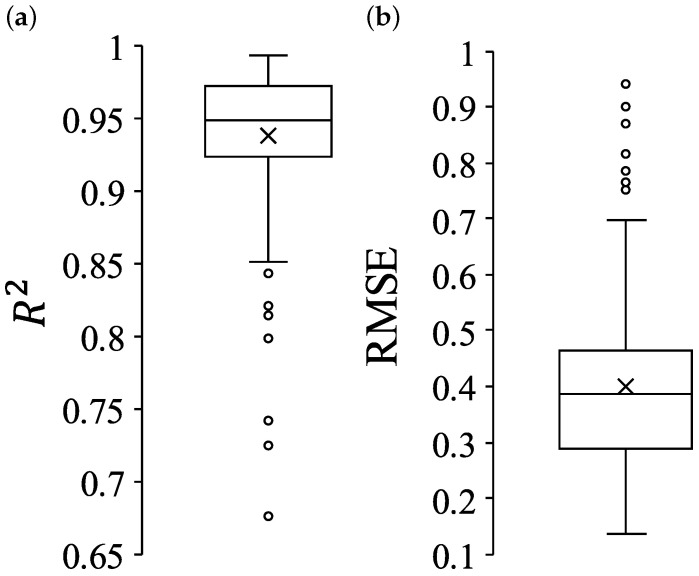
Box-whisker plots of (**a**) $R^{2}$ and (**b**) RMSE. The cross sign represents the arithmetic mean. The hollow circles are outlier samples. The number of samples was 160.

Figure 4 presents the box-whisker plots of $R^{2}$ and RMSE. In each plot, the whiskers extend to 1.5 times the interquartile range, serving as the upper and lower bounds. The cross sign represents the arithmetic mean, while hollow dots outside the whiskers indicate outliers. It is important to consider the full distribution of these indices, as some samples lie far from the center of the distribution.

## 4. Sensitivity Analysis

### 4.1. General Concept

In fields such as control engineering, the impact of input variables on the output is often explored through sensitivity analysis. Drawing on this concept, we can assess how each sensory attribute affects the temporal liking value. We propose two types of sensitivity analyses for this purpose.

The first type is elemental sensitivity, which measures the influence of an individual attribute on temporal liking, independent of other attributes. Analyzing elemental sensitivity allows us to determine which attribute has the most significant positive or negative effect on temporal liking.

The second type is interactive sensitivity, which evaluates how one attribute influences temporal liking in conjunction with another attribute, either synergistically or antagonistically. This analysis helps us understand whether the interaction between attributes enhances or diminishes their overall impact on temporal liking.

### 4.2. Definition of Sensitivities

**Figure 5:**
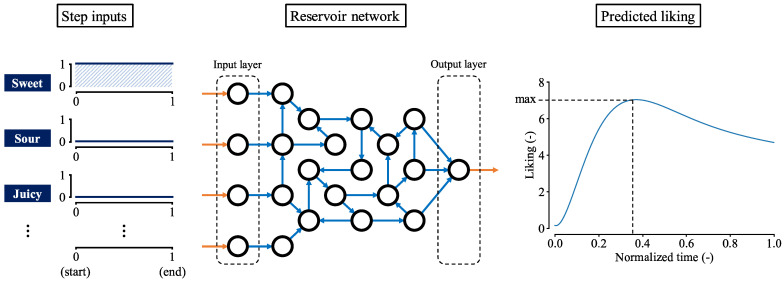
Example of step input and output. In this case, the dominance proportion of sweet is always 1 and those of the other attributes are 0. We give the step input to the reservoir model and record the maximum value of the output as y(sweet).

As shown in Figure 5, we provided the network model with TDS curves where the curve for a particular attribute remains at 1 for all t≥0, while the curves for the other attributes remain at 0. Here, *t* represents normalized time. We consider the model output for such an input corresponding to attribute *a* as its step response. The maximum value of this response, denoted as y(a), is defined as the elemental sensitivity of attribute *a*.

The maximum response for two attributes, denoted as y(a,b), is defined where the inputs for attributes *a* and *b* are held constant at 0.5 for all t≥0. Notably, y(a,a) is equal to y(a). We define the interactive sensitivity s(a) of attribute *a* as follows:

(1) $$\begin{array}{c} s\left(a\right)=\frac{1}{n-1}\sum_{b\in A}\left(y(a,b)-y(b)\right) \end{array}$$

where *n* is the number of attributes, and *A* is the set of all attributes, with $n=\left|A\right|$. If for some attribute *a*, y(a,b)=y(b) holds for all *b*, then s(a)=0. Conversely, if attribute *a* synergistically enhances the temporal liking in conjunction with other attributes, then y(a,b)>y(b) for some attributes *b*, resulting in a positive value for s(a). Thus, the interactive sensitivity quantifies the extent to which attribute *a* influences the effects of other attributes.

### 4.3. Sensitivities for Sensory Attributes of Strawberries

We calculated y(a,b) for all attribute combinations using 16 models. The averages of y(a,b), along with their 95% confidence intervals, are listed in Table 3. The diagonal elements represent the elemental sensitivities. Among all attributes, sweet and fruity exhibit the highest elemental sensitivities, indicating that these attributes are the most favorable for temporal liking. These are followed by the sensitivity for juicy. In contrast, the lowest values were observed for light and green, suggesting that these attributes are less effective in enhancing temporal liking.

The interactive sensitivities calculated from Table 3 are presented in Table 4. The corresponding *t*-values (paired *t*-test, two-tailed) indicate the statistical significance when compared to 0. The interactive sensitivities of green and light are significantly negative, suggesting that these two attributes tend to diminish the positive effects of other attributes.

## 5. Discussion

The reservoir network model developed in this study was able to predict temporal liking with an accuracy range of 67% to 99%. Approximately 5% of the predictions (7 out of 160 cases) exhibited lower accuracy. Reservoir networks hold the potential to reduce the cost and effort associated with sensory evaluations by predicting temporal liking curves from TDS curves. For instance, one practical application could be to predict the temporal liking curve for the TDS data of strawberries from a particular brand, using reservoir networks trained on data from other strawberry brands. However, the extent of this capability remains uncertain, and further investigation is needed to determine how well reservoir networks trained on similar types of foods perform when applied to other foods within the same category.

To implement the prediction of temporal liking scores and ensure robustness, two important steps should be followed. First, outliers in the TDS data should be removed, as discussed by previous studies [41]. Second, multiple models should be employed. In this study, 16 different models were generated, as described in Section 3 and Section 4. Despite using the same TDS curves as inputs, the output temporal liking curves varied slightly across models. Hence, it is advisable to use the mean and confidence intervals of these predicted curves to draw more reliable conclusions.

We introduced two types of sensitivities to quantify the influence of sensory attributes on liking: elemental sensitivity and interactive sensitivity. Elemental sensitivity measures how strongly each attribute affects liking on its own, while interactive sensitivity assesses how one attribute modifies liking when combined with others. Sweet and fruity were the attributes with the highest elemental and interactive sensitivities, indicating that they enhance temporal liking. In contrast, light and green showed the lowest sensitivities, suggesting that these attributes are less favorable for liking, a finding that aligns with previous studies [7,42,43].

According to Oliver et al. [42], sweet and fruity are key attributes of preferred strawberries, while green is associated with disliked strawberries. Okada et al. [7] reported that sweetness and a flavorsome quality contributed to high ratings for both liking and deliciousness. In the study by Lewers et al. [43], overall strawberry quality was strongly correlated with sweetness, as well as strawberry flavor and aroma. In this study, fruity was defined as the smell of sweet fruits, which closely aligns with the concept of strawberry aroma—that is, the fresh aroma characteristic of strawberries.

Interestingly, our results show that sourness, often considered a key attribute in the taste perception of fruits, did not strongly correlate with liking or overall evaluations of strawberries. This finding is consistent with the previous literature [7,42,43], where sourness was not found to be a primary determinant of strawberry preference. These results support the validity of the sensitivity analysis introduced in this study.

The sensitivity metrics proposed in this study indicate how each attribute of the TDS method influences the temporal liking score. Earlier researchers have also developed similar metrics, known as temporal drivers of liking. Thomas et al. [22] connected the TDS and temporal liking methods using a metric called LWD. The LWD value represents the mean temporal liking score while a certain attribute is selected in the TDS method. As a modification, the LWD values can be weighted by the duration during which the attribute is selected [24]. Meyners et al. [23,44] linked the liking scores and the temporal check-all-that-apply method using a penalty-lift analysis.

This raises a question: how are sensitivities and these indices for temporal drivers of liking qualitatively different? These indices are distinct in their computational methods; however, they may capture the effects of TDS attributes on temporal liking scores in a similar manner. A study comparing these metrics is suggested as a necessary future research direction. Such a comparison would provide valuable insights into the unique contributions and potential overlaps of these methods. Specifically, this comparison would allow us to explore criterion-related validity, as it would involve assessing the degree to which sensitivity metrics correlate with established measures of temporal liking. However, such an analysis requires extensive calculations and in-depth discussion and is therefore beyond the scope of this study. We propose to undertake this comparative analysis in future research to further substantiate the validity and added value of the sensitivity metrics introduced here.

The TDS and temporal liking methods capture the average preferences of all participating panels, which means that our approach is necessarily limited to average preferences. However, individual differences in preference are inevitable and are influenced by factors such as genetics, dietary habits, and age [45,46,47,48]. Predicting individual preferences through statistical or machine learning will likely require numerous additional steps. As a first step, it may be possible to classify a large number of individuals’ eating experiences and preferences into several groups [16,49,50]. For example, Louro et al. classified consumers into three groups based on a questionnaire about the frequency of consuming approximately 30 types of vegetables and fruits [50]. Developing group-specific models may help move closer to achieving predictions that account for individual differences.

One limitation of this study is that the sensitivity analysis only considered the maximum value of temporal liking, without accounting for the dynamic evolution of the TDS and liking curves over time. Future studies should incorporate time–series sensitivity analysis to evaluate how the influence of attributes changes throughout the consumption experience. For example, sourness might play a distinct role in the early or late stages of tasting.

Another limitation is that the reservoir network-based method was tested using only one type of food. To assess its broader applicability, this method should be applied to various food products. Additionally, further investigation is needed to determine how well a model trained on one brand of food performs when applied to other brands. While we hypothesize that a model developed for one strawberry brand may generalize to others, this assumption requires experimental validation. The same consideration applies to consumer groups; different estimation models may need to be developed for clusters with varying ages and food preferences.

## 6. Conclusions

This study represents the first attempt to predict temporal liking curves from TDS curves using reservoir computing. By modeling the relationship between TDS tasks and temporal liking tasks, we introduce a novel approach to linking two types of time–series sensory data. Through our analyses on strawberry data, the model demonstrated an estimation accuracy ranging from 67% to 99%, with a median of 95% and interquartile range of 92–97%. Additionally, we introduced two sensitivity measures: elemental sensitivity and interactive sensitivity. Elemental sensitivity quantifies the impact of each attribute on temporal preferences, while interactive sensitivity evaluates how one attribute influences liking in combination with others.

With further investigation into the general capabilities of the prediction model, its application in sensory evaluation could serve as a partial substitute for traditional evaluation procedures that rely on human assessors. Additionally, the prediction models would support advanced analyses, such as the sensitivity analysis introduced in this study. Future research should incorporate time–series dynamics into sensitivity analysis, as different attributes may have varying effects at different stages of food consumption. Furthermore, the proposed method should be validated across a variety of food types to assess its broader applicability.

## Figures and Tables

**Table 1:** Descriptions of attributes used in the TDS tasks. Adapted from [35].

Attribute	Description
Aromatic	Complex and pleasant smell.
Juicy	Amount of juice and flesh.
Sweet	Basic taste. No description was provided.
Fruity	Smell of sweet fruits.
Light	Sweet taste that does not last long in the mouth.
Watery	Water content with no strong taste.
Green	Smell, taste, and mouth feel of grass or unripe fruits.
Sour	Basic taste. No description was provided.

**Table 2:** Adjusted parameters for reservoir models.

Leaking rate	Spectral radius	Input scaling	Bias scaling
0.01	0.90	0.01–1.43	0.03–18.30
Input connectivity	Reservoir connectivity	Ridge regularization coefficient	
0.01–0.10	0.05–0.88	0.01–3.48	

**Table 3:** Averages and 95% confidence intervals of maximum outputs of temporal liking (y(a) and y(a,b) values) for all attribute combinations. Diagonal elements are elemental sensitivities y(a).

	Aromatic	Fruity	Green	Juicy	Light	Sour	Sweet	Watery
Aromatic	5.35 ± 1.49							
Fruity	6.26 ± 1.44	7.17 ± 2.09						
Green	4.71 ± 1.22	5.73 ± 1.21	4.46 ± 1.04					
Juicy	5.82 ± 1.19	6.88 ± 1.19	5.47 ± 1.16	6.44 ± 1.46				
Light	4.57 ± 1.13	5.52 ± 1.25	4.21 ± 0.87	5.23 ± 0.84	4.17 ± 0.99			
Sour	5.48 ± 1.16	6.57 ± 1.32	5.06 ± 0.74	6.25 ± 0.76	4.93 ± 0.74	5.78 ± 0.81		
Sweet	6.09 ± 0.98	7.11 ± 1.38	5.76 ± 0.41	7.09 ± 0.88	5.61 ± 0.74	6.75 ± 0.47	7.25 ± 0.84	
Watery	5.58 ± 1.04	6.66 ± 1.19	5.13 ± 0.76	6.22 ± 0.82	4.90 ± 0.83	5.95 ± 0.63	6.81 ± 0.69	6.04 ± 1.06

**Table 4:** Interactive sensitivities (means and 95% confidence intervals) of all attributes calculated from Table 3. *t* and *p* values were calculated for identifying the difference from 0. *p*-values were adjusted by Bonferroni correction of factor eight. The degrees of freedom of *t*-values are 15.

	Aromatic	Fruity	Green	Juicy	Light	Sour	Sweet	Watery
Sensitivity	−0.40 ± 1.04	0.75 ± 1.17	−0.88 ± 0.53	0.39 ± 0.91	−1.07 ± 0.56	0.02 ± 0.48	0.83 ± 0.68	0.09 ± 0.75
*t*-value	−1.078	1.906	−3.299	1.127	−3.955	0.064	2.779	0.293
*p*-value	1.00	0.61	0.036	1.00	0.007	1.00	0.11	1.00

## Data Availability

The original contributions presented in this study are included in the article. Further inquiries can be directed to the corresponding author.

## Abbreviations

The following abbreviations are used in this manuscript:

TDS	Temporal Dominance of Sensations
LWD	Liking While Dominant
